# Long-Acting Injectable Antipsychotic Treatment in Schizophrenia and Co-occurring Substance Use Disorders: A Systematic Review

**DOI:** 10.3389/fpsyt.2021.808002

**Published:** 2021-12-15

**Authors:** Alexandria S. Coles, Dunja Knezevic, Tony P. George, Christoph U. Correll, John M. Kane, David Castle

**Affiliations:** ^1^Centre for Complex Interventions, Centre for Addictions and Mental Health, Toronto, ON, Canada; ^2^Department of Psychiatry, University of Toronto, Toronto, ON, Canada; ^3^Department of Psychiatry and Molecular Medicine, Donald and Barbara Zucker School of Medicine at Hofstra/Northwell, Hempstead, NY, United States; ^4^Department of Child and Adolescent Psychiatry, Charité Universitätsmedizin Berlin, Berlin, Germany; ^5^Department of Psychiatry, The Zucker Hillside Hospital, Glen Oaks, NY, United States

**Keywords:** schizophrenia, substance use disorder (SUD), long acting injectable (LAI), antipsychotic, treatment

## Abstract

**Objectives:** Co-occurring substance use disorders (SUDs) among individuals with schizophrenia are a prevalent and complex psychiatric comorbidity, which is associated with increased symptom severity, worsened illness trajectory and high rates of treatment non-adherence. Recent evidence suggests that the use of long-acting injectable (LAI) antipsychotics may provide an effective treatment option for individuals with this dual-diagnosis.

**Methods:** A systematic review of the literature was conducted using the databases PubMed, PsychInfo and Google Scholar for English-language studies, investigating the use of LAIs in co-occurring schizophrenia and substance use disorders (SCZ-SUDs).

**Results:** Eight reports [one case study (*n* = 1), one case series (*n* = 8), three open-label retrospective studies (*n* = 75), and three randomized controlled trials (*n* = 273)] investigated the use of LAI antipsychotics in 357 participants with SCZ-SUDs [alcohol use disorder: 5 studies, *n* = 282; cocaine use disorder: 5 studies, *n* = 85; amphetamine use disorder: 1 study, *n* = 1; cannabis use disorder: 3 studies, *n* = 160; opioid use disorder: 3 studies, *n* = 19; methylenedioxymethamphetamine (MDMA) use disorder: 2 studies, *n* = 9; ketamine use disorder: 1 study, *n* = 4] and were included in this systematic review. Findings indicate significant improvements in substance use related outcomes across 7 of 8 studies, while in 6 of 8 studies, significant improvements in psychopathology-related outcomes were reported.

**Conclusions:** LAI antipsychotics may be an efficacious intervention option for the treatment of SCZ-SUDs. However, varying methodological rigor, generally small sample sizes and heterogeneity of samples, settings, substances of abuse, tested LAIs and comparators, as well as psychosocial cotreatments and level of reported detail across studies requires that these findings be considered preliminary and interpreted with caution. Further research is required to better understand the effects of LAIs among individuals with SCZ-SUDs.

## Introduction

Schizophrenia (SCZ) and co-occurring substance use disorders (SUDs) present a prevalent and clinically complex comorbidity (referred to hereafter as SCZ-SUDs) that significantly worsens illness trajectory and is associated with increased morbidity and mortality (1, 2). Approximately 40–65 percent of individuals with schizophrenia also have a co-occurring SUD, with cannabis, alcohol and stimulants representing the most commonly misused substances (2). Persistent misuse of alcohol and drugs by this population is associated with several adverse consequences, including increased rates of homelessness, incarceration, and suicide (2). Moreover, SCZ-SUDs has been linked to increased burden for emergency healthcare services, greater service utilization and higher rates of hospitalization (3). Patients with this dual diagnosis often experience worsened cognitive and negative symptoms, more frequent positive symptoms, higher rates of depression and relapse, and a less stable illness course, than those without such comorbidity (4, 5). Research in this domain points to SUDs as a major barrier to functional recovery among individuals with schizophrenia (4). Additionally, treatment adherence within this population is remarkably low: the SCZ-SUDs comorbidity is associated with reduced therapeutic engagement, as well as high rates of oral medication non-adherence, representing additional barriers to successful treatment and a need for long term solutions (6). The pervasive impact of SCZ-SUDs combined with these complicating factors frame an urgent requirement to develop effective treatment options to improve outcomes for individuals with this comorbidity.

### Traditional Treatments for SCZ-SUDs

Psychosocial approaches have been studied for treatment of individuals with SCZ-SUDs, including motivational interviewing and enhancement, relapse prevention training, and cognitive behavioral therapy. A meta-analysis by Bennett et al. (4) found that these psychosocial interventions are associated with moderate efficacy in this population, particularly for improvements in SUD related outcomes such as abstinence or use reductions. However, psychosocial treatments are not recommended as sufficient treatments alone for SCZ-SUDs but should be used in conjunction with pharmacotherapy as a multi-faceted approach to treatment (4).

In terms of medications, there is a scant and inconsistent literature for comorbid SCZ-SUDs. There are two broad (and non-exclusive) psychopharmacological approaches to treatment in this group of patients: (1) the use of antipsychotic medications (e.g., risperidone, clozapine) to improve psychotic symptoms, which may also target mechanisms relevant to SUDs; (2) the use of antipsychotic medications in combination with anti-craving or anti-use agents (e.g., disulfiram, naltrexone). A large-scale systematic review by Azorin et al. (7) evaluated the evidence for oral antipsychotic medication treatment in individuals with SCZ-SUDs from 152 treatment studies. Based on direct and indirect evidence, findings were in support of second-generation (serotonin-dopamine antagonist) rather than first-generation (dopamine antagonist) antipsychotics in this population. Specifically, for individuals with comorbid cocaine use disorder, olanzapine and haloperidol were associated with improvements in both psychiatric and SUD outcomes in several studies (8, 9). For cannabis use disorder, clozapine and ziprasidone were superior, providing improvements in both psychiatric and SUD outcomes (10–12). Finally, olanzapine and quetiapine were most successful in the treatment of SCZ and alcohol use disorder. Regarding SUD-specific medications, results indicate that both naltrexone and disulfiram may be successful in reducing alcohol intake among individuals with schizophrenia and alcohol use disorder (13, 14). Additionally, the tri-cyclic antidepressants imipramine and desipramine were helpful in reducing cocaine craving and use in patients with co-occurring schizophrenia and cocaine use disorder (7). However, authors emphasized that evidence to support these recommendations is limited and should be considered preliminary. There is a critical need for further controlled research in this area, though preliminary indications are promising.

### Long-Acting Injectables (LAIs)

A major barrier to successful treatment of SCZ-SUDs remains the low rate of treatment adherence. LAI antipsychotics, one of the most effective psychiatric interventions available for people with schizophrenia, are traditionally used as maintenance therapy in chronic schizophrenia and may be an effective treatment option for SCZ-SUDs while providing a viable solution to improvement of adherence issues in this population (15).

LAI antipsychotics (also known as depot antipsychotics) are injectable formulations of medications that release the active drug slowly (weeks to months, depending on the formulation) (16). Several studies have investigated the efficacy of LAI antipsychotics among individuals with schizophrenia compared to placebo, with positive results: A network meta-analysis by Ostuzzi et al. (17) of 78 RCTs (*n* = 11,505) indicated that most of the twelve meta-analyzed LAIs outperformed placebo regarding relapse prevention, except for some older first-generation LAIs (i.e., Haloperidol, Bromperidol, Zuclopenthixol and Flupenthazine). For acceptability, most LAIs outperformed placebo, being associated with significantly less all-cause discontinuation (17). In a separate meta-analysis, Kishimoto et al. (18) compared LAI antipsychotics to oral antipsychotics across three different designs; there were 137 studies encompassing 397,319 patients with schizophrenia (i.e., 32 randomized controlled trials (RCTs) [23.4%; *n* = 8577], 65 cohort studies [47.4%; *n* = 377,447], and 40 mirror-image studies [29.2%; *n* = 11,295]). Across all three designs, LAIs were associated with a significantly lower risk of hospitalization or relapse than oral antipsychotics [RCTs: RR = 0.88 (95% CI = 0.79–0.99), *p* = 0.033; cohort studies: RR = 0.92 (0.88–0.98), *p* = 0.0044; mirror image studies: RR = 0.44 (0.39–0.51), *p* < 0.0001]. Across all other outcomes related to effectiveness, efficacy, safety, quality of life, cognitive function, and other outcomes, LAIs were more beneficial than oral antipsychotics in 60 (18.3%) of 328 comparisons, not different in 252 (76. 8%) comparisons, and less beneficial in 16 (4.9%) comparisons (mostly driven by unequal antipsychotic type in the LAI and oral antipsychotic group, leading to adverse effect differences). A separate meta-analysis of tolerability and safety outcomes specifically compared the same LAI and oral antipsychotics in RCTs: LAI formulations demonstrated similar rates of adverse effects in 115 of 119 reported adverse effects, including extrapyramidal symptoms, suggesting they are safe and well tolerated therapeutic options (19).

In addition to superior efficacy and effectiveness with LAIs vs. oral antipsychotics and similar safety and tolerability, including rare cases of neuroleptic malignant syndrome where LAIs cannot be stopped abruptly (20–22) there are a number of potential further benefits to using LAI formulations. Primarily, as LAIs are administered every 2 weeks to 3 or, even, 6 months-depending on medication and formulation (15, 16)-patients experience both a reduced pill burden and are more likely to adhere to treatment (23). Additionally, as LAIs require clinician administration, a more realistic understanding of adherence to treatment is possible, and an enhanced therapeutic alliance can ensue. Individuals taking LAI antipsychotics have also described an improved quality of life compared to those taking oral formulations (15). LAIs have greater bioavailability than oral agents, due to their bypassing liver degradation at first-pass metabolism, allowing for greater available drug concentrations in the central nervous system (23). LAI antipsychotics further have a more reliable delivery system, maintaining steady drug plasma levels and eliminating the peak to trough concentration related side effects common with oral antipsychotics.

In sum, LAI antipsychotics are effective, safe, and tolerable in individuals with schizophrenia, as well as demonstrating considerable potential benefits over oral formulations, notably in terms of adherence. Thus, LAIs may provide a feasible treatment option for individuals with SCZ-SUDs. The current article is a systematic review and critical evaluation of studies investigating the efficacy of LAIs as treatments in SCZ-SUDs.

## Methods

A thorough review of the available literature was conducted by two independent reviewers (A.C. & D.K.) employing the following four databases: PubMed, PsychInfo, Cochrane and Google Scholar. The search strategy followed the Cochrane's PICOS framework for systematic reviews (**P**articipants, **I**ntervention, **C**omparator, **O**utcomes and **S**tudy Design). The following key search terms were used in varying combinations to identify relevant articles: (Schizophreni* OR Schizoaffective OR Psychosis OR psychotic) AND (Substance use OR Substance Dependence OR Substance Use Disorder OR Substance abuse OR Substance Misuse OR Cocaine OR Alcohol OR Amphetamine* OR Opioid* OR opiate* OR Heroin or Cannabi* OR phencyclidine OR ketamine OR psychedelic* OR multisubstance OR polysubstance OR NPS OR “novel psychoactive”) AND (Long-Acting Injectable Antipsychotic* OR Long Acting Injectable OR Depot OR Intramuscular OR flupenthixol OR fluphenazine OR Zuclopenthixol OR Haloperidol OR Aripiprazole OR Risperidone OR Paliperidone) AND (Open-Label OR Randomized Controlled Trial OR Retrospective or Observational OR Qualitative OR Prospective). Articles to be included in this systematic review had to meet the following eligibility criteria:

### Inclusion Criteria

- Articles published in peer-reviewed, English-language journals
- The use of both a psychopathology related, and substance use related outcome measure
- Adult participants with schizophrenia-spectrum disorders and co-occurring substance use disorders (alcohol use disorder, cocaine use disorder, cannabis use disorder, amphetamine use disorder, stimulant use disorder, opioid use disorder)
- The use of long-acting injectable antipsychotic treatment as the primary intervention
- All study designs accepted

This systematic review was conducted in accordance with Cochrane's *Preferred Reporting of Systematic Reviews and Meta Analyses* (PRISMA) Guidelines.

## Results

### Study Selection and Characteristics

**Figure 1:**
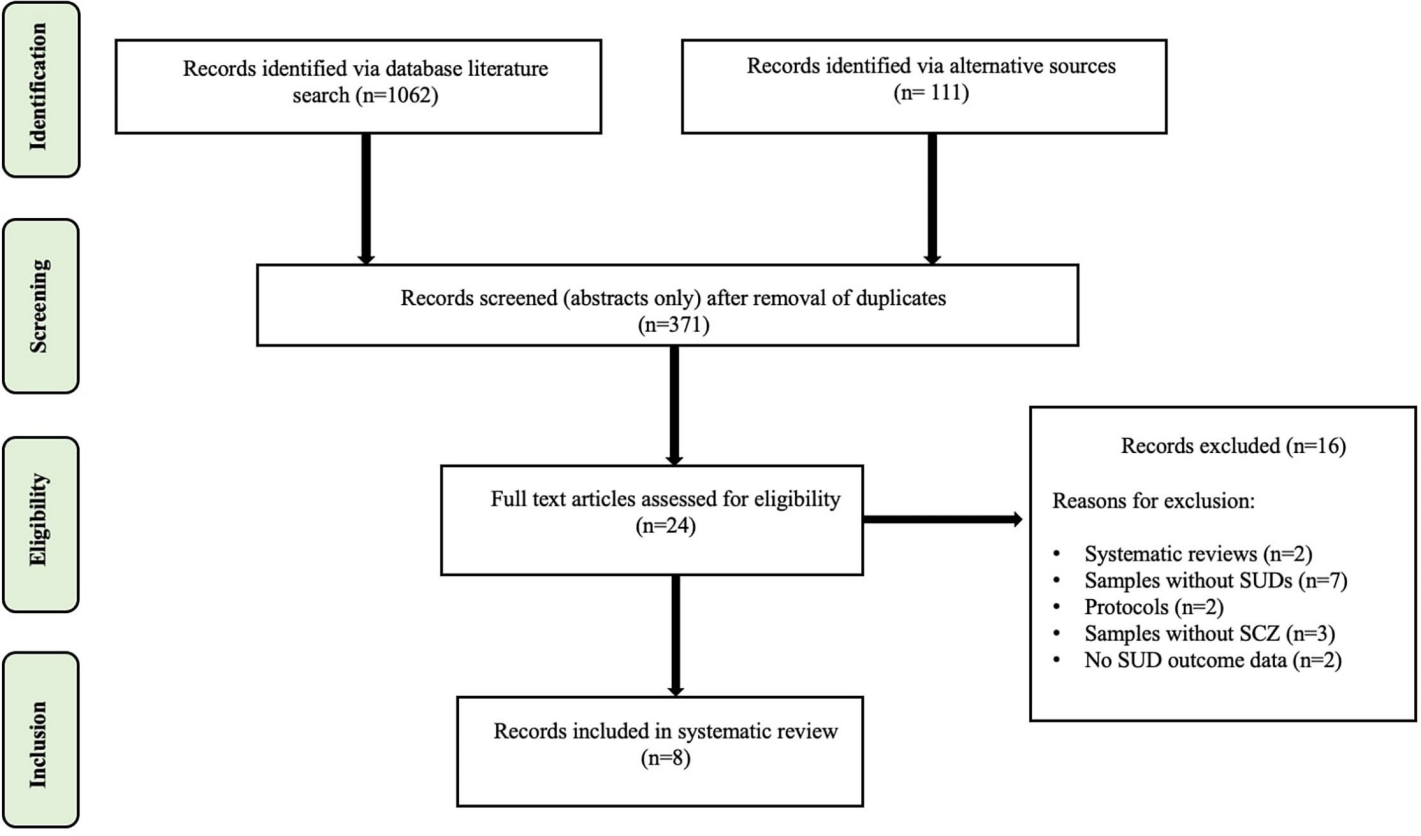
Prisma consort diagram.

The search strategy identified 1,602 articles from the four databases (PubMed, PsychInfo, Cochrane, Google Scholar) and a further 111 articles were identified through other methods (i.e., ClinicalTrials.gov, reference lists of similar review articles, etc.) (see Figure 1). After the removal of duplicates, a total of 371 articles remained eligible for abstract review, of which 24 were eligible for full text analysis. Of these 24 articles, 16 were excluded, i.e., 7 due to not including samples with SUDs, three that did not include individuals with schizophrenia-spectrum disorders, and 2 reports each due to being systematic reviews, study protocols, or studies that did not report on SUD outcomes. This left 8 articles meeting complete inclusion criteria that were included for analysis, in alignment with PICOS protocol.

### Results Synthesis

Eight reports [one case study (*n* = 1), one case series (*n* = 8), three open-label retrospective studies (*n* = 75), and three randomized controlled trials (*n* = 273)] investigated the use of LAI antipsychotics in 357 participants with schizophrenia and comorbid SUDs [alcohol use disorder: 5 studies, *n* = 282; Cocaine use disorder: 5 studies, *n* = 85; amphetamine use disorder: 1 study, *n* = 1; cannabis use disorder: 3 studies, *n* = 160; opioid use disorder: 3 studies, *n* = 19; methylenedioxymethamphetamine (MDMA) use disorder: 2 studies, *n* = 9; ketamine use disorder: 1 study, *n* = 4] and were included in this systematic review (see Table 1 for study summaries).

**Table 1:** Long acting injectable antipsychotics for comorbid schizophrenia and substance use disorders.

**Total studies** **=** **8, Total** ***N*** **=** **395**					
**Study**	**Sample**	**Study design**	**Intervention**	**Results**	**Effect size (Cohen's** ***d*****)**
**Case studies (*****n*** **=** **2 studies**, ***n*** **=** **9 participants with SSD)**					
Ouhuha et al. (24)	*N* = 9 participants with SCZ (*n* = 8) or BP (*n* = 1) and Cocaine use disorder	A naturalistic case series	HPD-IM or FLX-IM (5–15 mg oral equivalents per day)	No significant effects on psychopathology or substance use symptoms were observed.	^*^
Chen et al. (25)	*N* = 1 participant with SCZ and Amphetamine use disorder	Case study	LAI-AP (400 mg/4 weeks)	Significant reduction in psychotic symptoms and cravings for amphetamines were observed.	^*^
**Open-label trials (*****n*** **=** **3 studies**, ***n*** **=** **75 participants with SSD)**					
Levin et al. (26)	*N* = 8 participants with SSD and Cocaine use disorder	A 10-week open-label trial	FLX-IM (40 mg/2 weeks)	A 28 percent reduction in cocaine-positive urine screens, though most patients had a reduction of > 75 percent. Marked reductions in SCZ and depression symptoms were observed across participants.	^*^
Soyka et al. (27)	*N* = 27 participants with SSD and Alcohol use disorder	An open-label exploratory multicenter 6-month trial	LAI-FLX (10–60 mg)	Significant reductions in alcohol use were observed across participants (8 participants were abstinent at study termination). Minimal improvements in psychopathology were recorded.	Psychopathology outcome: Pre vs. Post-treatment scores LAI-FLX: *d* = 0.35 Substance use outcome: Pre vs. Post-treatment scores LAI-FLX: *d* = 0.99
Szerman et al. (28)	*N* = 40 participants with SSD and one or more SUDs (Alcohol, *n* = 16; Cannabis, *n* = 17; Opioids, *n* = 4; Cocaine, *n* = 9) (# of poly substance users not disclosed)	A multicenter, naturalistic, observational, retrospective study	LAI-AP (*n* = 31, 400 mg/month; *n* =5, 300 mg/month; *n* = 3, 400 mg/ 3 weeks; *n* = 1, 400 mg/2 weeks)	A 30% reduction in psychotic symptom severity scores were observed across participants. No significant effects on substance dependence severity, apart from cocaine and alcohol. Alcohol use change from 10.6 (3.9) at baseline to 8.9 (3.2) at follow-up. Cocaine use change from 11.2 (4.9) at baseline to 8.4 (3.5) at follow-up.	Psychopathology outcome: Pre vs. Post-treatment scores LAI-AP: *d* = 2.34 Substance use outcome: Pre vs. Post-treatment craving scores Alcohol subgroup: *d* = 0.48 Cocaine subgroup: *d* = 0.66
**Randomized controlled trials (*****n*** **=** **3 studies**, ***n*** **=** **273 participants with SSD)**					
Rubio et al. (29)	*N* = 115 participant with SCZ and one or more comorbid SUDs (Alcohol, *n* = 101; Cannabis, *n* = 82; Cocaine, *n* = 30; Opioids, *n* = 10; MDMA, *n* = 5) (# of poly substance users not disclosed)	A randomized, controlled, 6-month follow-up study	LAI-RP (n.d.; *n* = 57) or ZP depot (n.d.; *n* = 58)	Participants who received LAI-RP saw significantly greater clean urine screens compared to ZP depot (*P* = 0.005), as well as greater improvements in symptom severity on the PANSS	Psychopathology: Post-treatment scores LAI-RP vs. ZP-depot: *d* = 0.45 Positive urine screens: Post-treatment scores LAI-RP vs. ZP-depot: *d* = 0.52
Green et al. (30)	*N* = 95 participants with SCZ and Alcohol use disorder	A randomized controlled trial	LAI-RP (25 mg titrated to 37.5 mg/2weeks: *n* = 49) or Oral risperidone (4 mg/day; *n* = 46)	No significant SCZ symptom differences between groups. Heavy drinking worsened in the oral risperidone group. LAI-RP saw significantly less heavy drinking days per week compared to oral risperidone (*p* = 0.035).	^*^
**Study**	**Sample**	**Study design**	**Intervention**	**Results**	**Effect size (Cohen's** ***d*****)**
Cuomo et al. (31)	*N* = 101 inpatients with SSD (*n* = 63), FEP (*n* = 27) or BP (*n* = 11) and one or more SUDs (Alcohol, *n* = 43; Cannabis, *n* = 61; Cocaine, *n* = 30; MDMA, *n* = 4; Ketamine, *n* = 4; Opioids, *n* = 5) (*n* = 34/101 were polysubstance users)	A randomized controlled trial	LAI-AP (400 mg/ 4 weeks; *n* = 50) or LAI-PP (100 mg/4 weeks)	Both groups saw significant reductions in clinical symptoms and substance related cravings, as well as improved quality of life. AP, compared to PP, maintained craving and quality of life improvements at 1-year follow up.	Psychopathology: Pre vs. Post-treatment scores LAI-AP: *d* = 6.26 LAI-PP: *d* = 4.74 Craving Intensity: Pre vs. Post-treatment scores LAI-AP: *d* = 4.08 LAI-PP: *d* = 1.31

* *Data was insufficient or not available for calculation of effect sizes. LAI, Long-Acting Injectables; SCZ, Schizophrenia; SSD, Schizophrenia Spectrum Disorders; AP, Aripiprazole; FEP, First Episode Psychosis; PP, Paliperidone; BP, Bipolar Disorder; RP, Risperidone; AUD, Alcohol Use Disorder; n.d., No Dose; ZP, Zuclopenthixol; PANSS, Positive and Negative Symptom Scale; FLX, Flupentixol; IM, Intramuscular; HPD, Haloperidol*.

#### Case Studies and Case Series

A naturalistic case series of eight individuals with schizophrenia and cocaine use disorder treated with haloperidol decanoate or flupenthixol decanoate, was reported by Ouhuha et al. (24). LAI use was not associated with any improvements in either psychotic symptoms or cocaine use. No information on safety and tolerability were reported (24).

A case study by Chen et al. (25) reported a 26-year-old female with schizophrenia and amphetamine use disorder who was treated with 400 mg of LAI-aripiprazole every 2 weeks. This patient reported significant decreases in positive and cognitive symptoms related to schizophrenia, as well as a significant reduction in amphetamine craving. No objective measures of symptom change were included. At 1-year follow up, this participant reported achieving abstinence from amphetamines, which was further confirmed by multiple negative urine toxicology screens. Maintenance of improved psychopathology was also reported at 1-year. No information regarding safety or tolerability of medications were indicated (25).

#### Non-randomized Studies

Three non-randomized, open-label, retrospective studies (*n* = 75) have been conducted to investigate the use of LAI antipsychotics in SCZ-SUDs. In the first, Levin and colleagues (26) investigated the use of flupenthixol decanoate in eight patients with schizophrenia-spectrum disorders, with a focus on comorbid cocaine use disorder. This study entailed two-phases: participants began the study in a 4-week inpatient phase, followed by a 6-week outpatient phase. Upon study initiation, participants were cross tapered off current antipsychotic medications, and commenced on oral flupenthixol (maximum oral dose of 12 milligrams per day) for a period of 6 days, before being switched to the decanoate version, beginning at 20 milligrams IM /week. All participants were encouraged to attend group psychoeducation and life skills sessions on a weekly basis during the outpatient phase of the trial. Significant reductions in severity of psychopathology were observed across participants at all time points post baseline (*p* < 0.05). Notably, overall changes in cocaine-positive urine screens were not statistically significant, though five of eight participants showed a trending decline in positive urine screens from baseline to follow up. Moreover, participant ratings of cocaine craving were substantially reduced over time, though statistical significance was not reached, probably due to the low statistical power of the study. Study medications were safe and well tolerated by participants (26).

Soyka et al. (27) conducted an open-label relapse prevention trial in 27 people with schizophrenia and comorbid alcohol use disorder. Participants were treated with 10–60 mg of flupenthixol decanoate (mean dose of 30.4 mg) every 2 weeks for a period of 24 weeks. All participants in the intent-to-treat sample consumed at least 120–150 milliliters of pure alcohol daily at baseline. Fourteen participants (66.6%) completed the study, with main reasons for premature termination reportedly due to adverse effects related to study medication (i.e., severe akathisia) or poor adherence with study procedures. At study termination, 8 of 14 participants (57.1%; 38.1% of the total enrolled) were abstinent from alcohol, and an additional two reported significant reductions in use compared to baseline. In participants who did not achieve abstinence, mean drinks per day were reduced from 7.7 (+/- 5.8) to 4.4 (+/- 3.2) (*d* = 0.99). Finally, craving scores, as measured by the *Obsessive-Compulsive Drinking Scale (*OCDS) decreased significantly between visit one and two for all participants and remained at this reduced level for the entirety of the study. Regarding changes in psychopathology between baseline and 6 months (post-treatment), 50% of participants were categorized as much improved or very much improved, whilst 21% reported no change or worsened severity of psychopathology at study termination (*d* = 0.35). Nine of 27 participants experienced at least one adverse effect, though study medications were generally well tolerated by participants. Notably, extrapyramidal symptoms were minimal (27).

A recent multicentre, retrospective observational study was conducted by Szerman et al. (28) to determine the efficacy of 400 mg per month of LAI-aripiprazole in forty participants with SCZ-SUDs. Results from this 6-month descriptive study showed that treatment with LAI-aripiprazole was associated with clinically significant reductions in psychopathology severity from baseline–determined by a>30 percent reduction in scores on the *CGI-S*–for 77.5% of participants (*d* = 2.34). Mean scores on the *WHODAS* (a measure of disability) also decreased significantly (*M* = 57.6, *SD* = 8.2, to M = 42.3, *SD* = 4.3). Substance use changes were most significant in individuals with cocaine use disorder and alcohol use disorder, with 5 of 9 and 3 of 16 participants, respectively, achieving abstinence by the end of the study. All three participants with heroin use disorder were abstinent at 6 months follow-up. Further, scores on the *Severity of Dependence Scale* (SDS) for individuals who did not achieve abstinence within these substance use categories showed significant reductions: cocaine [from *M* = 11.2 (4.9) to *M* = 8.4 (3.5), *d* = 0.66], and alcohol [from *M* = 10.6 (3.9) to *M* = 8.9 (3.2), *d* = 0.48] (all *p's* < 0.001). Data on safety and tolerability of LAI-aripiprazole was not reported (28).

#### Randomized Controlled Trials

Three of the included studies were RCTs, encompassing a total of 273 individuals with SCZ-SUDs. In two studies, two LAIs were compared head-to head, and in one RCT an LAI was compared to the same antipsychotic (risperidone), given orally. The earliest of these was conducted by Rubio and colleagues (29) as a 6-month follow up study in 115 participants with schizophrenia and SUDs (alcohol: *n* = 101, cocaine: *n* = 30, cannabis: *n* = 82, opioids: *n* = 10 or MDMA: *n* = 5). Participants were randomized to receive open-label LAI-risperidone (47.2 mg/15 days + 2–6 mg/day of oral risperidone) or zuclopenthixol-depot (200 mg/21 days + 10–50 mg/day of oral zuclopenthixol) over the course of 6 months. Participants also attended weekly substance use training sessions, which were based on the *Substance Abuse Management Model* (SAMM) of Roberts et al. (32). Significant improvements in psychopathology (measured by the Positive and Negative Symptom Scale for Schizophrenia, *PANSS*) were observed in both treatment groups, though LAI-risperidone was superior: 89% of those on risperidone had a reduction of at least 20% on the PANSS (general scale) vs. 50% in the zuclopenthixol-depot group (*d* = 0.45) (*p* < 0.001). Substance use changes were measured as a function of clean urine screens in the weeks following treatment initiation. Individuals in the LAI-risperidone group had a significantly greater number of clean urine screens and a longer time to relapse (first relapse took place in week 9) than the individuals in the LAI-zuclopenthixol group (first relapse took place in week 7) (*d* = 0.52). Additionally, adherence was higher in the LAI-risperidone group, with a greater number of participants also attending the substance use management training sessions, compared to the LAI-zuclopenthixol group. Finally, both LAI-risperidone and LAI-zuclopenthixol were well tolerated by study participants. Notably, there were significantly less extrapyramidal effects observed in the LAI-risperidone group, while antiparkinsonian drugs were used more often in the LAI-zuclopenthixol group, suggesting that LAI-risperidone may be more tolerable in this population (29).

A second randomized trial, by Green et al. (30) compared the efficacy of LAI vs. oral risperidone in 95 participants with schizophrenia and co-occurring alcohol use disorders over a 6-month period. Participants were titrated to a mean dose of 4.3 mg per day in the oral risperidone group, or a mean dose of 32.7 mg every 2 weeks in the LAI-risperidone group. Explanatory analyses indicated that heavy drinking significantly worsened in the oral group over the study period (average increase of 0.68 heavy drinking days per week), though not in the LAI-risperidone group (average decrease in heavy drinking days−0.011) (*p* = 0.24). No differences between groups were observed in drinking intensity (days of drinking per week). Additionally, no differences in symptom severity (measured by the PANSS) were found post-treatment in either group. Treatment adherence was significantly lower in the oral risperidone group compared to the LAI group. Finally, safety, tolerability and side effect profiles were similar for both the oral and LAI-risperidone groups, with a total of 79% of all participants experiencing an adverse event during the study (30).

Finally, Cuomo et al. (31) conducted a comparison of two LAI antipsychotic medications in 125 inpatient participants with a diagnosis of either schizophrenia or bipolar disorder (with psychotic features) and a comorbid SUD (alcohol: *n* = 43, cannabis: *n* = 61, cocaine: *n* = 30, MDMA: *n* = 4, opioids: *n* = 5 and ketamine: *n* = 4). Participants were randomized to receive either 400 mg of intramuscular aripiprazole monohydrate or 100 mg intramuscular paliperidone palmitate once per month, for a period of 12 months. Significant improvements across measured outcomes from baseline to follow up (1-year) were observed for both groups. Specifically, LAI-aripiprazole and LAI-paliperidone were both associated with improved symptom severity (based on *Clinical Global Impressions Scale, CGI*) with large effect sizes of *d* = 6.26 and *d* = 4.74, respectively (*p*'s < 0.001). Further, LAI-aripiprazole was superior to LAI-paliperidone in the reduction of substance-related craving intensity, though both groups showed significant improvements in this domain (*d* = 4.48 and *d* = 1.31, respectively) (*p*-value < 0.001). Notably, two participants in the LAI-paliperidone group reported increased craving post-treatment. This result is of particular interest, as baseline values indicated stronger craving intensity in participants allocated to the LAI-aripiprazole group. Additionally, both medications had significant improvements in quality of life, though effect sizes for LAI-aripiprazole were much larger than those for LAI-paliperidone (*d* = 1.98 and *d* = 0.65, respectively) (*p*-value < 0.001). Few side effects were reported, of which none led to study discontinuation. Side effects were less in the LAI-aripiprazole group compared to the LAI-paliperidone group, demonstrating similar side effect profiles and tolerability as their oral formulations. Five patients in the LAI-paliperidone group did develop hyperprolactinemia, of whom four also developed galactorrhea. Finally, two participants in the LAI-aripiprazole group developed akathisia, leading to a reduction of dose from 400 to 300 mg, which eliminated the side effect in both participants. Study related changes in weight were not reported (31).

## Discussion

The current article is a systematic review of available studies (case reports, case series, open-label studies, and randomized controlled trials) assessing the efficacy of LAI antipsychotics for the treatment of schizophrenia and co-occurring SUDs.

A single case report (25) observed a positive outcome for LAI-aripiprazole treatment in a woman with schizophrenia and co-occurring amphetamine use disorder, while a small-scale case series showed no benefit for LAI-flupenthixol or LAI-haloperidol in comorbid schizophrenia and cocaine use (24). While instructive, case series and case reports are inevitably subject to reporting bias, and thus, little can be concluded from these studies.

The three open-label studies reported in this review (26–28) are aligned in terms of apparent efficacy of LAIs for psychotic symptoms and indices of substance use (specifically, alcohol and cocaine). However, all studies involved small samples, were of retrospective design and were limited in duration. Moreover, the different psychotropic agents could not be compared with one another. Alone, these studies do not allow any firm conclusions to be drawn regarding the efficacy of the LAIs themselves (i.e., over, and above simple inclusion in the study).

The three randomized controlled trials included in this review (29–31) allow for the comparison of either LAI vs. oral antipsychotics or the comparison across different LAIs. Green et al. (30) found that LAI-risperidone was associated with better alcohol-related outcomes on some indices, compared to oral risperidone. This study lends support to the use of LAIs in people with schizophrenia who also have alcohol use disorder and underscore the benefits of assured adherence in this population.

The study by Rubio et al. (29) compared a first-generation antipsychotic, LAI-zuclopenthixol, with a second-generation agent, LAI-risperidone. It is of note that outcomes with LAI-risperidone were somewhat superior, as it has been suggested that the second-generation antipsychotic LAIs have improved tolerability compared to the older agents (33). Further, the review by Azorin et al. (7) suggested that some of the second-generation antipsychotics may have advantages over the older, first-generation medications in terms of efficacy for people with schizophrenia and a comorbid SUD. In terms of a comparison between LAI antipsychotic agents (i.e., aripiprazole monohydrate and paliperidone palmitate), Cuomo and colleagues (31) observed similar efficacy of both agents in the treatment of psychotic symptoms, though aripiprazole had stronger anti-craving effects in SCZ-SUDs.

Notably, none of the reviewed randomized controlled trials included a placebo condition. Though this can be defended based on clear evidence for the efficacy of antipsychotics (and LAIs in particular) in reducing the risk of relapse in people with schizophrenia (17), the absence of placebo-controlled studies limits the interpretation of results.

In general, all study medications in LAI form were considered safe and well tolerated by study participants. This aligns with previous research that has demonstrated similar side effect profiles and risk of adverse events and extrapyramidal symptoms for both LAI and oral formulations of antipsychotic medications (15, 19).

### Strengths and Limitations

This systematic review was conducted in accordance with internationally accepted guidelines for systematic reviews (PRISMA and PICOS guidelines) and contains a broad range of all available literature on the use of LAIs in SCZ-SUDs.

There are some limitations to the current review, as well as methodological limitations of reviewed studies, which must be highlighted. A wide range of study designs were deliberately included, given the paucity of trials in the area. Despite this broad set of inclusion criteria, our yield was modest, and the studies were highly heterogeneous, precluding a meta-analysis.

Regarding methodological limitations, the reviewed studies employed a variety of LAI medications at different doses and at varying dose intervals (as determined by the particular product), making comparisons across studies problematic. Also, a wide variety of different substances of abuse were included, with many of the larger studies including participants who simultaneously abused a number of substances: alcohol, cannabis, opioids, cocaine and MDMA in the study of Rubio et al. (29) and those agents in addition to ketamine in the study of Cuomo et al. (31). Of the RCTs, only that of Green et al. (30) included people using only one substance (i.e., alcohol). It is thus difficult to draw conclusions about LAI efficacy in patients with specific drugs of abuse.

The types of participants included in the reviewed studies were generally later in their illness course, which emphasizes the gap in understanding the early use of LAI antipsychotics in people with emerging psychosis and SUDs. This is a pertinent problem, given the various guidelines, which call for judicious use of LAIs earlier in illness course (i.e., first episode psychosis) [e.g., (34, 35)]; and compelling data for their efficacy in such individuals, including from the recent PRELAPSE study (36).

Length of follow-up also varied significantly, ranging from a few weeks to 12 months. Arguably, the proof of efficacy and safety of LAIs is determined via maintenance of effects in the years of follow-up. Thus, only the randomized trial conducted by Cuomo et al. (31) is of sufficient length for meaningful clinical conclusions to be drawn about longer-term use, and longer-term trials are of critical need.

Most sample sizes were small and did not have sufficient statistical power to allow analyses of sub-groups, a notable issue due to the heterogeneity of substances of abuse included (see above). The study settings also varied, ranging from inpatient to community environments, or a combination of the two. Finally, concomitant psychosocial interventions also varied across studies, ranging from none (or not specified) to adjunct use of an established efficacious psychosocial intervention for SUDs (29).

## Conclusions

Substance use disorders are common among people with schizophrenia and have been shown to worsen the longitudinal course of illness, reduce medication adherence and increase rates of relapse. The fact that a number of LAI second generation antipsychotics show efficacy and good tolerability for people with schizophrenia and are associated with enhanced adherence and reduced relapse rates, suggests they deserve special consideration in people with SCZ-SUDs. The evidence reviewed here supports this assertion, but the paucity of studies and methodological shortcomings temper this conclusion. The sparsity of available literature on the subject speaks to the difficulties in conducting research in populations with comorbid substance use problems, who are often specifically excluded from clinical trials. Given the prevalence of comorbid substance use in individuals with severe mental illness, further research in this area is urgently required.

## Data Availability Statement

The original contributions presented in the study are included in the article/supplementary material, further inquiries can be directed to the corresponding authors.

## Author Contributions

AC and DK performed literature review. AC wrote first draft of the manuscript. AC and DC wrote sections of the manuscript. All authors contributed to the revision and approval of the submitted manuscript.

## Conflict of Interest

CC has been a consultant and/or advisor to or has received honoraria from: AbbVie, Acadia, Alkermes, Allergan, Angelini, Aristo, Axsome, Damitsa, Gedeon Richter, Hikma, Holmusk, IntraCellular Therapies, Janssen/J & J, Karuna, LB Pharma, Lundbeck, MedAvante-ProPhase, MedInCell, Medscape, Merck, Mitsubishi Tanabe Pharma, Mylan, Neurocrine, Noven, Otsuka, Pfizer, Recordati, Relmada, Rovi, Seqirus, Servier, SK Life Science, Sumitomo Dainippon, Sunovion, Supernus, Takeda, Teva, and Viatris. He provided expert testimony for Janssen and Otsuka. He served on a Data Safety Monitoring Board for Lundbeck, Relmada, Rovi, and Teva. He has received grant support from Janssen and Takeda. He received royalties from UpToDate and is also a stock option holder of LB Pharma. DC has received grant monies for research from Eli Lilly, Janssen Cilag, Roche, Allergen, Bristol-Myers Squibb, Pfizer, Lundbeck, Astra Zeneca, Hospira; Travel Support and Honoraria for Talks and Consultancy from Eli Lilly, Bristol-Myers Squibb, Astra Zeneca, Lundbeck, Janssen Cilag, Pfizer, Organon, Sanofi-Aventis, Wyeth, Hospira, Servier, Seqirus; and is a current or past Advisory Board Member for Lu AA21004: Lundbeck; Varenicline: Pfizer; Asenapine: Lundbeck; Aripiprazole LAI: Lundbeck; Lisdexamfetamine: Shire; Lurasidone: Servier; Brexpiprazole: Lundbeck; Treatment Resistant Depression: LivaNova; Cariprazine: Seqirus. He is a founder of the Optimal Health Program OHP, currently operating as Optimal Health Australia, which holds the IP for OHP; is part owner of Clarity Healthcare. The remaining authors declare that the research was conducted in the absence of any commercial or financial relationships that could be construed as a potential conflict of interest.

## Publisher's Note

All claims expressed in this article are solely those of the authors and do not necessarily represent those of their affiliated organizations, or those of the publisher, the editors and the reviewers. Any product that may be evaluated in this article, or claim that may be made by its manufacturer, is not guaranteed or endorsed by the publisher.
